# A Near-Miss: Arrow Trauma to the Skull Without Penetration

**DOI:** 10.7759/cureus.89204

**Published:** 2025-08-01

**Authors:** Diego Ángeles-Sistac, Luis E García-San Jose, Esteban Bermeo-Jarrin, Jose I Pérez-García

**Affiliations:** 1 Critical Care Medicine, Hospital de la Santa Creu i Sant Pau, Barcelona, ESP; 2 General Surgery, Hospital de la Santa Creu i Sant Pau, Barcelona, ESP

**Keywords:** arrow injury, computed tomography, emergency department, emergent general surgery, head and neck trauma, neuro-critical care

## Abstract

We report the case of a 77-year-old woman who presented to the emergency department (ED) after being unexpectedly struck in the head by an arrow while walking through a densely populated urban area in Barcelona, Spain. This highly unusual presentation in a modern city context prompted immediate multidisciplinary evaluation in the ED. Initial radiographs showed a metallic arrow tip in close proximity to the skull’s cortical surfaces. Non-contrast head computed tomography (CT) and 3D reconstruction confirmed a left parietal soft tissue injury without intracranial penetration. The patient underwent a successful removal of the arrow under local anesthesia, with no complications, and was discharged without neurological deficits.

This case highlights the critical role of advanced imaging and multidisciplinary collaboration in the management of cranial arrow injuries, even in densely urbanized settings. Continued case reporting is essential to inform and support the development of evidence-based approaches for these rare but potentially life-threatening events.

## Introduction

Arrow injuries to the head are exceptionally rare in contemporary urban environments [1-8], as most penetrating head and neck trauma is more commonly caused by gunshot wounds [6]. Arrow injuries are more frequent in developing countries and rural settings, where most victims are men, and the incidents are often the result of interpersonal violence or conflict [1-8].

In contrast, cranial arrow trauma in developed countries is exceedingly uncommon and typically results from accidental incidents involving recreational archery [5,9-12]. On rare occasions, these injuries are linked to suicide attempts, most often involving crossbows [7,9]. Although arrows are classified as low-velocity projectiles, at close range, they can produce penetrating trauma comparable to that of a low-powered handgun [8].

Due to their rarity and the limited use of advanced imaging in reported cases, detailed radiological documentation of such injuries, particularly in urban settings, is scarce. Consequently, standardized treatment approaches in the emergency department (ED) for managing these cases in major city hospitals are often lacking [9].

While the general approach should follow protocols for traumatic brain injury (TBI), arrow wounds present unique considerations. These include the identification of the arrow type (e.g., serrated, barbed, or toxin-coated) and meticulous surgical planning, as arrow removal is often the most technically challenging step [9]. Arrows should not be removed blindly; depending on the arrowhead design, pushing it along its original trajectory may be safer than extraction in reverse [8].

Despite the low incidence of arrow-related head injuries, the increasing recreational use of bows in Western countries, combined with the high morbidity and mortality rates associated with penetrating brain trauma (estimated between 34% and 93%), underscores the importance of detailed case reporting and literature review [9,11].

We report the case of a 77-year-old woman who was unexpectedly struck in the head by an arrow while walking through the city of Barcelona, Spain. The incident occurred at night in a typical urban neighborhood when a boy and his father, practicing recreational archery in their garden, accidentally released an arrow that struck the victim. The unusual nature of the event drew nationwide media attention [13,14]. Upon arrival at the ED, a fast-acting multidisciplinary team, supported by advanced imaging techniques, enabled an accurate diagnosis and guided a successful, minimally invasive intervention.

## Case presentation

A 77-year-old woman with a medical history of hypertension and dyslipidemia presented to the emergency department (ED) after being struck in the head by an arrow while walking through a typically urban area in Barcelona, Spain. The arrow, consistent with those used recreationally in archery competitions, was embedded in the left parietal region. The origin of the projectile was unknown at the time of presentation.

Upon the arrival of emergency services, the patient was alert and oriented, with a Glasgow Coma Scale (GCS) score of 15. She had no focal neurological deficits but reported headache and dizziness. Vital signs were stable: Blood pressure was 168/77 mmHg, heart rate was 60 beats per minute (bpm) in sinus rhythm, and she was eupneic with an oxygen saturation of 100% on ambient air. Primary examination revealed no additional injuries.

The arrow was stabilized with bandages, and the patient was treated with 150 mcg of intravenous fentanyl and 2 mg of granisetron. She was transported to the ED without neurological or hemodynamic deterioration. Initial venous blood gas analysis was unremarkable, with a lactate level of 1.2 mmol/L.

**Figure 1:**
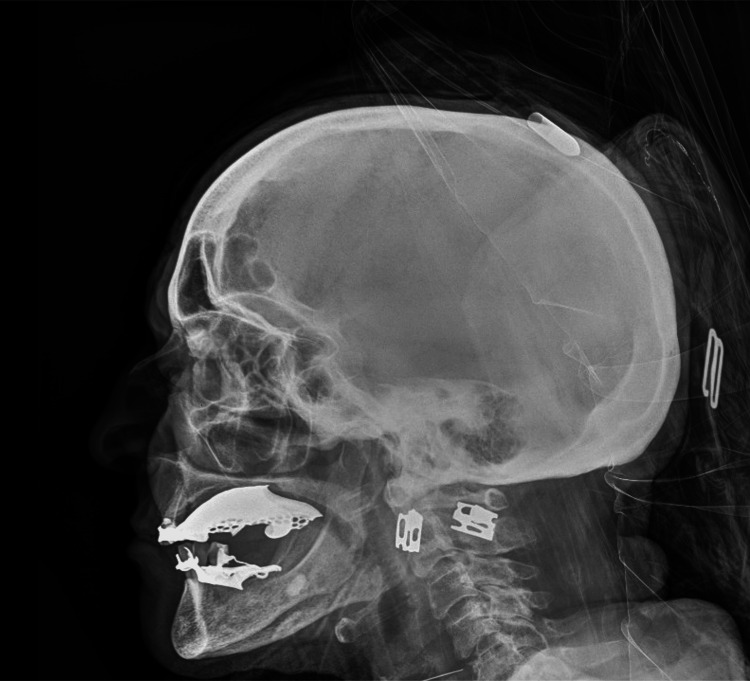
Lateral cranial X-ray. The arrow tip seems apparently lodged between the outer and inner cortical tables of the skull, without evidence of intracranial penetration.

**Figure 2:**
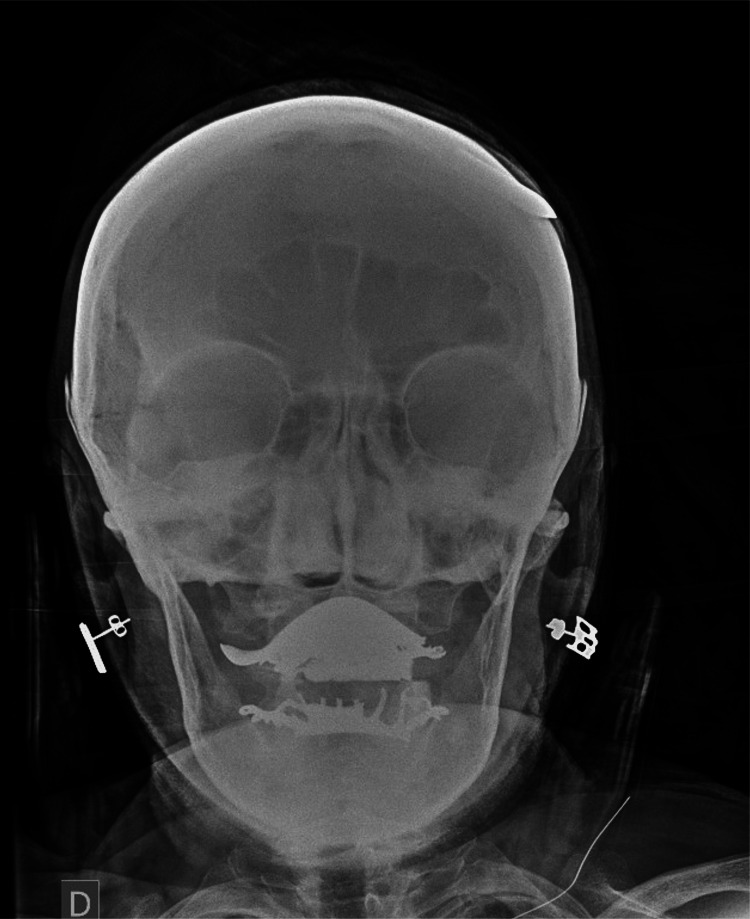
Frontal cranial X-ray. The arrow tip in apparent contact with or partially penetrating the outer cortical bone of the skull.

**Figure 3:**
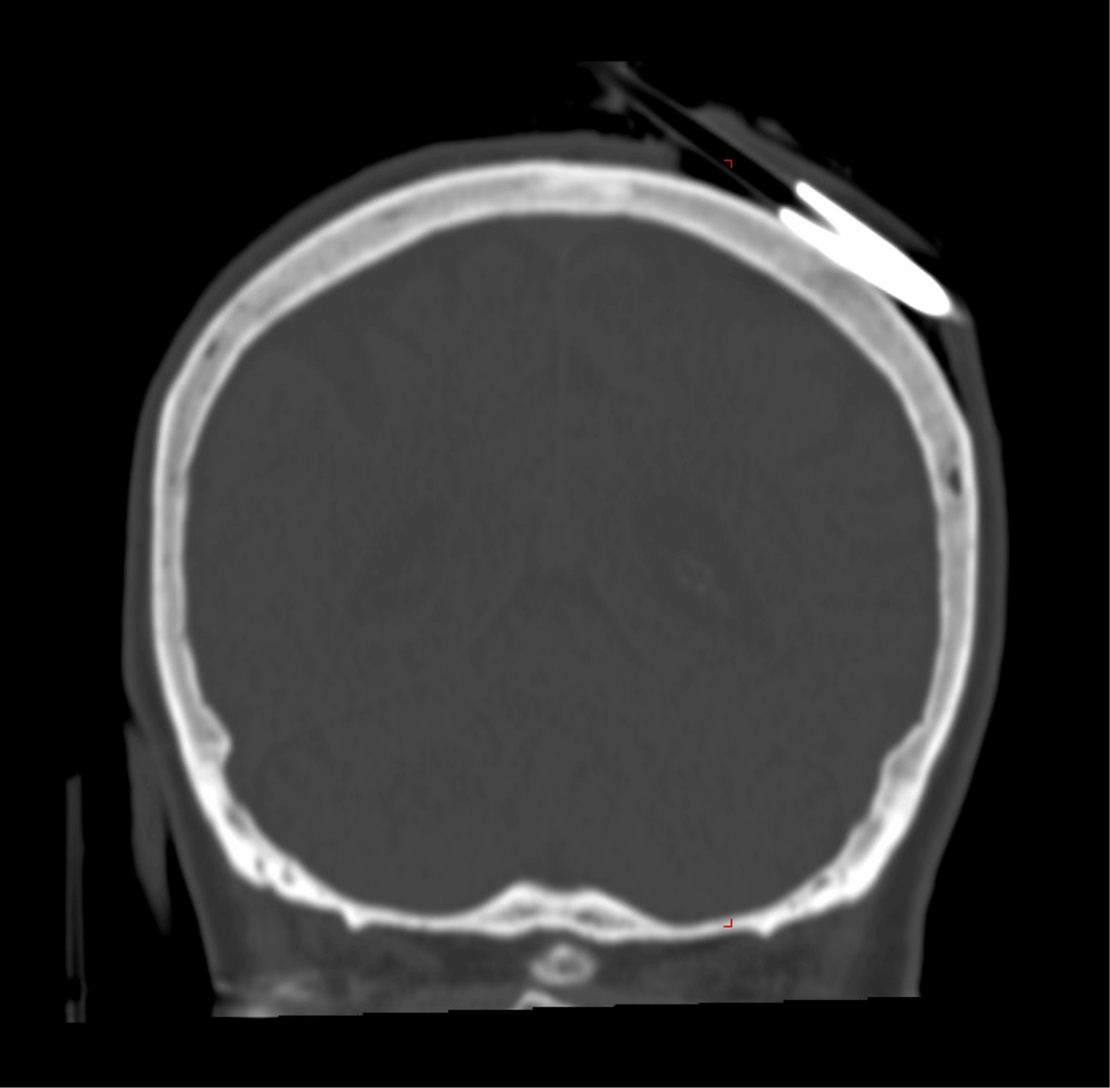
Coronal head CT. Soft tissue injury over the left parietal region with the arrow tip in contact with the external cortical bone, without evidence of intracranial involvement. CT: computed tomography

**Figure 4:**
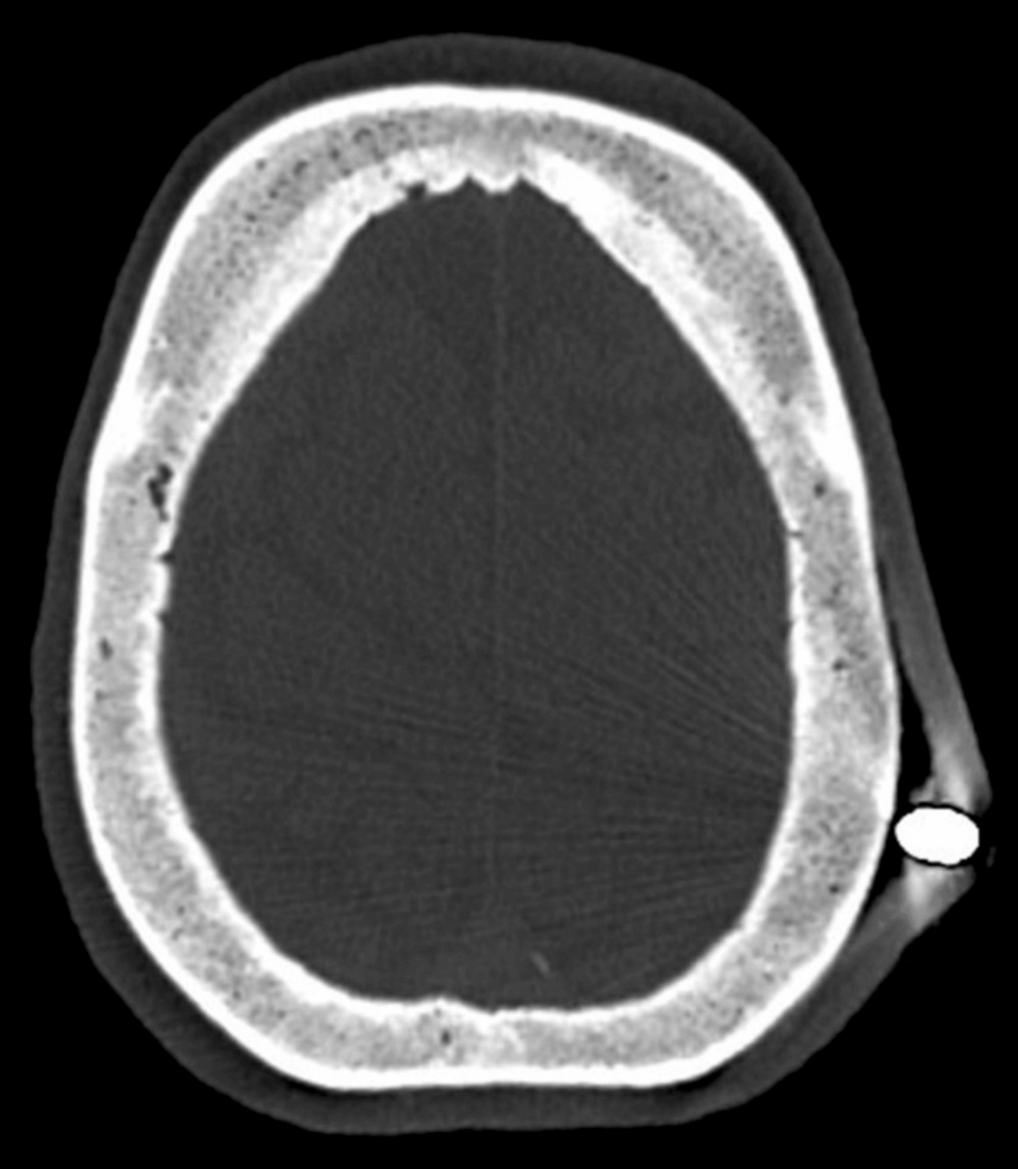
Axial head CT. Soft tissue injury over the left parietal region with the arrow tip in contact with the external cortical bone, without evidence of intracranial involvement. CT: computed tomography

**Figure 5:**
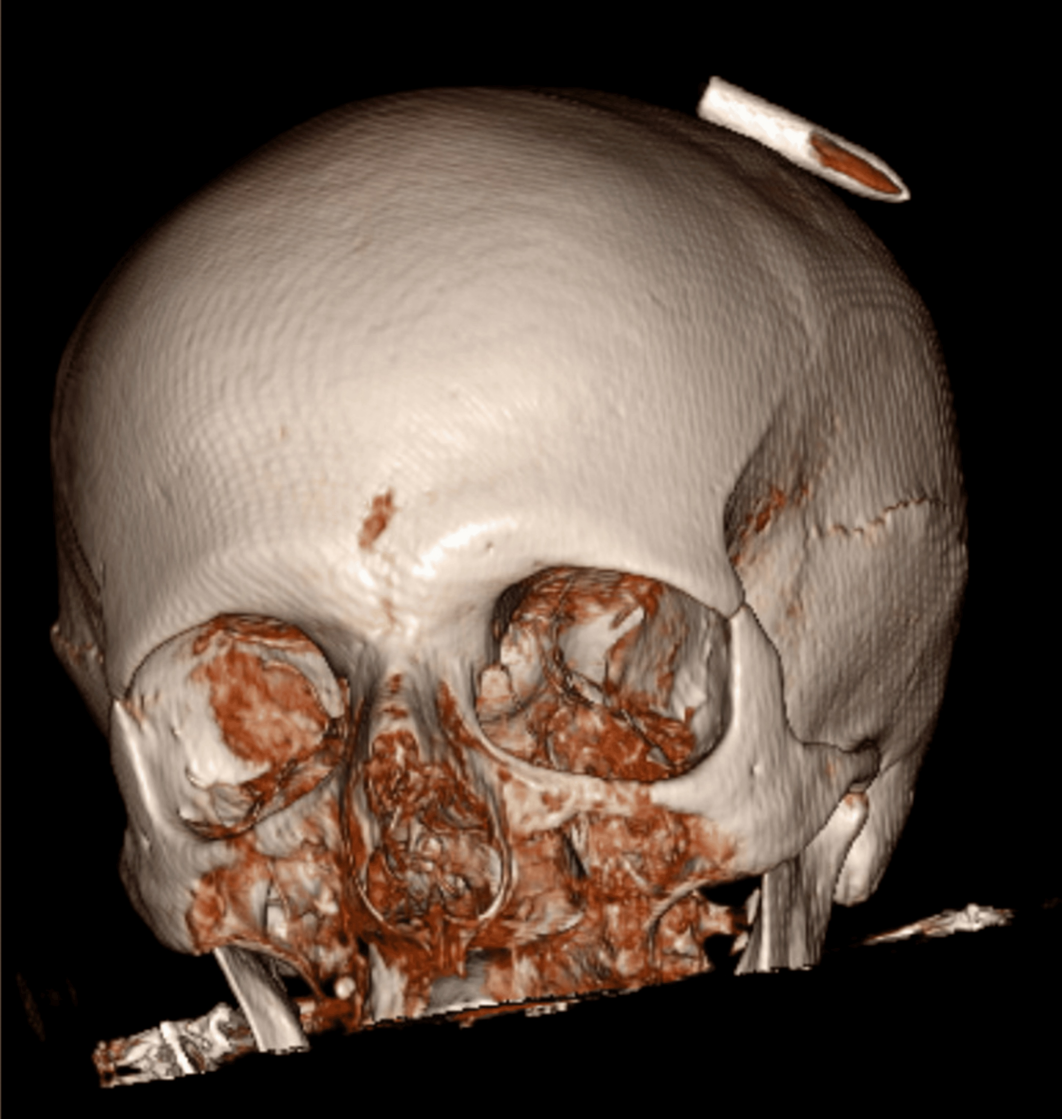
3D head CT reconstruction. The arrow tip is positioned just above the external cortical bone, with no evidence of intracranial involvement. CT: computed tomography

Frontal and lateral cranial radiographs (X-rays) revealed a metallic arrow tip in close proximity to, or potentially lodged between, the outer and inner cortical tables of the skull (Figure 1), without definitive evidence of cranial vault penetration (Figure 2). Since the extent of the lesion was not clear, a subsequent non-contrast head computed tomography (CT) confirmed a left parietal soft tissue injury caused by the arrow (Figure 3), with the tip abutting the calvarium but no evidence of intracranial involvement (Figure 4). A 3D CT reconstruction further illustrated the superficial trajectory and location of the arrow (Figure 5).

The patient was evaluated by the neurosurgery team. Given that the arrowhead was conical and continuous with the shaft, without barbs or serrations, it was safely removed in reverse. The wound was sutured under local anesthesia in the operating room without complications. Tetanus prophylaxis was administered, and due to the superficial nature of the injury, antibiotic prophylaxis was not deemed necessary. At 24 hours post admission, the patient remained neurologically intact and free of complications related to the injury and was subsequently discharged. Follow-up at two years revealed that the patient had fully recovered and exhibited no sequelae.

## Discussion

Arrow injuries to the head, sometimes referred to as “William Tell injury,” are rare in the modern era, particularly in urban areas of developed countries [1-5]. The few cases that have been reported typically originate from rural or tribal regions in developing countries, often resulting from interpersonal conflict [1-8].

In contrast, arrow-related cranial injuries in Western countries are uncommon and are usually associated with suicide attempts, particularly involving crossbows [1,3]. Injuries caused by conventional bow-and-arrow use are even rarer and, when documented, are typically the result of accidental incidents during recreational activities [5,10,11]. Even in these scenarios, such injuries most often occur in open fields or designated shooting areas, rather than in densely populated urban settings [5,10,11]. The rarity and geographical clustering of these cases limit the availability of detailed radiological documentation.

Although arrows are classified as low-velocity projectiles, they can still be life-threatening, particularly when fired at close range or when they impact vital structures, such as in head or neck injuries [4,8,12]. The extent of injury depends on several factors, including firing distance, the degree of penetration, impact force, arrowhead type, bow tension, projectile mass, and whether the tip was coated with toxins or poisons [5,7-9,12].

The management of cranial arrow injuries is not yet standardized, but a multidisciplinary approach is strongly recommended [2-4,11]. This includes coordination among emergency medicine, surgery, neurosurgery, and critical care teams. General treatment principles should mirror those of TBI and include infection prevention (e.g., prophylactic antibiotics and tetanus immunization), intracranial pressure management, and measures to prevent secondary injury [5].

Specific considerations for arrow injuries include leaving the arrow in situ during transport and initial imaging. Any manipulation prior to full evaluation may risk worsening tissue damage [2-4,6,9,12]. The assessment of potential injury during extraction is critical, as retrieval can exacerbate damage to surrounding tissues [2-4,6,9,12].

When available, CT, particularly with 3D reconstruction, offers the most comprehensive evaluation, especially in hemodynamically stable patients [1,2,4,9,12]. If there is evidence of intracranial penetration, urgent cerebral angiography is advised to assess for vascular injury [5]. Once metallic pieces are removed or discarded, magnetic resonance imaging (MRI) may also be useful, especially for detecting retained wood fragments [6].

Unconventional arrowheads, such as barbed, serrated, or fanged tips, pose additional challenges due to their increased risk of iatrogenic injury during extraction [2,4]. For this reason, the arrow should always be left in situ until appropriate imaging and surgical planning have been completed [1,4]. When available, obtaining an identical arrowhead can aid in preoperative planning [5]. In select cases, removal along the original trajectory, rather than withdrawal in reverse, may reduce the risk of further tissue damage [5].

## Conclusions

This case illustrates a highly unusual presentation of cranial arrow trauma in an elderly woman walking through a modern European city, an event rarely documented in the literature. Despite the low velocity of the projectile, the potential for life-threatening injury is significant when vital structures are involved. Fortunately, in this case, the arrow tip did not breach the cranial vault, allowing for conservative surgical management and a favorable clinical outcome.

The case underscores three critical aspects in the management of penetrating cranial injuries of this nature. First, coordination between the ED, general surgery, neurosurgery, and intensive medicine was crucial for a safe and timely intervention. Second, advanced imaging, particularly CT with 3D reconstruction, was essential for assessing the depth and trajectory of the lesion, which informed the surgical strategy. Third, the importance of maintaining the arrow in situ until full diagnostic evaluation is complete cannot be overstated, as premature manipulation may lead to secondary injury. In light of the growing popularity of archery as a recreational activity in urban settings, EDs must be prepared to recognize and manage such rare but potentially serious injuries. The continued reporting of these cases will help develop standardized management approaches and improve outcomes.
